# Case Report: A Canonical Splice-Site COL4A5 Variant in Alport Syndrome in a Kazakhstani Family

**DOI:** 10.3390/cimb48060588

**Published:** 2026-06-02

**Authors:** Diana Basharova, Ayazhan Bekbayeva, Gulnara Svyatova, Aizhan Darmeshova, Elena Zholdybayeva

**Affiliations:** 1 National Center for Biotechnology, Astana 010000, Kazakhstan; dbasharova451@gmail.com (D.B.); bekbayeva@biocenter.kz (A.B.); 2 Center for Molecular Medicine, Almaty 050000, Kazakhstan; gsvyatova1@mail.ru; 3 LLP «Algamed», Almaty 050000, Kazakhstan; adilaizhan87@mail.ru

**Keywords:** Alport syndrome, XLAS, *COL4A5*, WES, intron variant, splice-site prediction, in silico analysis

## Abstract

Background: Alport syndrome is a hereditary disorder caused by defects in the type IV collagen network. Although exon variants are primarily associated with Alport syndrome, the clinical significance of intronic variants remains incompletely characterized. The aim of this study was to characterize the clinical and molecular features of a familial case of Alport syndrome associated with the intronic variant c.1588-2A>G and to assess its impact using in silico tools. Case description: Two affected siblings presented with hematuria, proteinuria, and renal biopsy demonstrated focal global and segmental glomerulosclerosis, findings consistent with Alport syndrome. Whole-exome sequencing was subsequently performed in patients. The variant (NM_033380.2, c.1588-2A>G) in intron 23 of the *COL4A5* gene was identified in both probands. SpliceAI analysis demonstrated a complete loss of the canonical acceptor site and a high probability of cryptic site activation. Conclusion: The evidence suggests a likely pathogenic role of the *COL4A5* c.1588-2A>G variant in Alport syndrome.

## 1. Introduction

Alport syndrome (AS) is a hereditary disorder characterized by progressive kidney damage, sensorineural hearing loss, and eye abnormalities [1]. AS is caused by defects in the type IV collagen network, which is a major structural component of the basement membranes of the kidneys, inner ear, and eyes [2]. The *COL4A3*, *COL4A4*, and *COL4A5* genes encode the α3, α4, and α5 chains of type IV collagen, respectively. AS results from a genetic abnormality in one of the three chains [3]. Large deletions affecting both *COL4A5* and *COL4A6* are associated with more complex phenotypes, including Alport syndrome with diffuse leiomyomatosis (AS-DL) [4].

AS is classified into three subtypes based on the mode of inheritance: X-linked Alport syndrome (XLAS), autosomal recessive AS (ARAS), and autosomal dominant AS (ADAS). XLAS is caused by pathogenic variants in the *COL4A5* gene, which encodes the α5 chain of type IV collagen, while ADAS and ARAS are caused by variants in the *COL4A3* or *COL4A4* genes, which encode the α3 or α4 chains of type IV collagen, respectively [5]. XLAS is the most common type of AS, accounting for approximately 80% of all AS cases [1]. A significant number of variants were detected in patients with XLAS, with missense variants being the most common (approximately 38.0%), followed by deletion (15.9%) and splice variants (approximately 14.9%) [3,6].

There is a significant correlation between genotype and phenotype in males with XLAS. Specifically, individuals with missense or non-truncating variants exhibit less severe phenotypes compared with those with truncating variants. Furthermore, patients with splice-site variants exhibit intermediate phenotype severity, falling between those associated with non-truncating and truncating variants [2]. Intronic variants are particularly important in the pathogenesis of hereditary diseases, including Alport syndrome, which is caused by abnormalities in the *COL4A5* gene. Pathogenic variants in this gene are the main cause of the X-linked variant of the disease, with a significant proportion of them affecting splicing [5].

The prevalence of Alport syndrome varies from one in 5000 to one in 53,000 individuals [7]. Historically, a diagnosis required strict clinical criteria, including characteristic kidney biopsy findings, sensorineural hearing loss, ocular abnormalities, persistent hematuria, and renal dysfunction, often accompanied by a positive family history. However, recent expert consensus guidelines recommend genetic confirmation as mandatory, defining Alport syndrome more broadly to include all individuals with pathogenic variants in the *COL4A3*, *COL4A4*, or *COL4A5* genes, regardless of their clinical presentation. Although molecular technologies, such as direct sequencing and targeted next-generation sequencing (NGS), are increasingly used due to their diagnostic reliability and noninvasiveness, these methods have limitations. Notably, they typically do not detect variants located in deep intronic or regulatory regions, potentially leading to missed diagnoses [8].

To identify deep intronic variants, transcriptional analysis, such as RNA sequencing, is essential. However, amplifying *COL4A5* transcripts extracted from peripheral blood leukocytes presents considerable challenges. Thus, extracting mRNA from urine sediment is recommended due to the ease of obtaining urine samples and the presence of cells that express mRNA for all three genes. In addition to transcript analysis using mRNA from patient samples, functional in vitro splicing analysis (minigene analysis) using an expression vector is also useful, as it can be used to identify potential splicing variants in *COL4A5* [2].

Canonical splice-site variants (CSSVs) are DNA variants that affect splice donor (+1 and +2) and acceptor (−1 and −2) sites, which define the boundaries of exons and introns. The consensus nucleotide sequences at splice donor and acceptor sites are GT and AG, respectively, and they are required for interaction with the U2 spliceosome, leading to normal splicing and the generation of wild-type transcripts. CSSVs can alter the interaction between the mRNA precursor and the spliceosome complex. The resulting splicing events can include exon skipping, complete intron inclusion, and alternative use of nearby cryptic splice-sites, resulting in nucleotide insertions or deletions (indels). These effects may or may not cause a frameshift and a premature stop codon, which can then trigger nonsense-mediated RNA degradation and lead to a loss of gene function [9].

The aim of this study was to characterize the clinical and molecular impact of the *COL4A5* c.1588-2A>G splice-site variant in two pediatric patients from Kazakhstan with suspected Alport syndrome, using an integrated approach that combines clinical phenotyping, in silico prediction, and protein structural modeling.

Clinical and whole-exome sequencing (WES) findings from this family were previously reported by Basharova et al. in Astana Medical Journal (2025) as part of a study of children with suspected Alport syndrome [10]. However, the pathogenic role and molecular consequences of the *COL4A5* c.1588-2A>G splice-site variant were not investigated in detail in the previous publication. In the present report, the variant was additionally confirmed by Sanger sequencing and comprehensively evaluated using ACMG/AMP criteria, SpliceAI prediction, and structural modeling approaches.

## 2. Case Description

**Figure 1:**
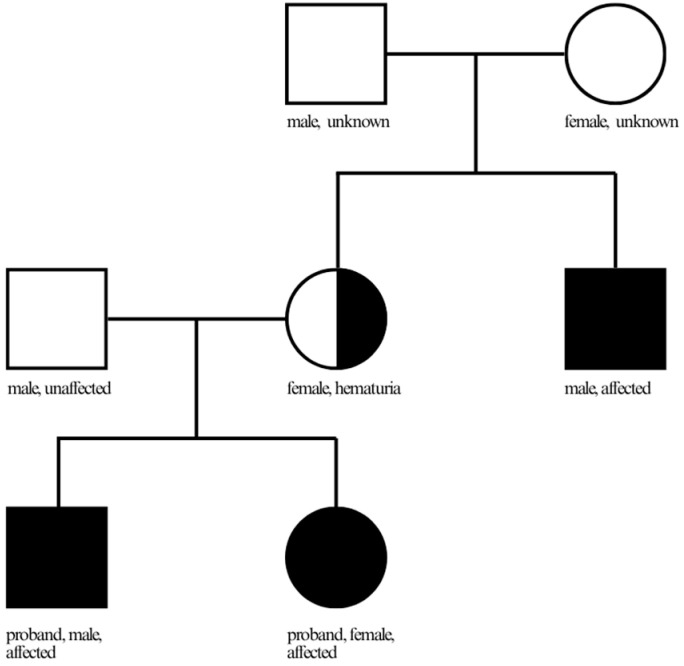
Pedigree of the family. Affected individuals are indicated by filled black symbols, half-filled symbols indicate individuals with hematuria, and open symbols represent unaffected or phenotypically unknown family members.

We report two related patients from the same family from Kazakhstan presenting with clinical manifestations consistent with Alport syndrome. Both individuals are siblings with a positive family history. Their mother has hematuria, and their maternal uncle reportedly had a similar renal disease requiring dialysis at the age of 30 years. Further clinical and genetic information regarding relatives of patients was unavailable. The family pedigree is shown in Figure 1.

Patient 1 is an 8-year-old female examined for persistent urinary abnormalities. The onset of the disease was noted at the age of 6 years, when routine laboratory testing revealed microscopic hematuria (10–15 erythrocytes per high-power field) and proteinuria (0.33 g/L). The initial clinical diagnosis was hereditary nephritis with chronic kidney disease stage G1A2. No sensorineural hearing loss or ophthalmologic abnormalities were detected. Renal ultrasonography revealed bilateral thickening of the walls of the pelvicalyceal system. Histopathological examination of a renal biopsy specimen demonstrated focal global and segmental glomerulosclerosis, accompanied by interstitial fibrosis and tubular atrophy of grade 1. Electron microscopy findings were consistent with a hereditary defect of type IV collagen.

Laboratory tests showed the following values in the complete blood count: hemoglobin 131 g/L, erythrocytes 4.93 × 10^12^/L, platelets 365 × 10^9^/L, leukocytes 6.71 × 10^9^/L, and erythrocyte sedimentation rate (ESR) 4 mm/h. Urinalysis revealed clear, straw-colored urine with a specific gravity of 1.003 and a pH of 6.5; leukocytes were detected at 2/μL, and erythrocytes at 80/μL. Biochemical blood analysis showed total protein 61 g/L, ALT 10.6 U/L, AST 21 U/L, urea 3.5 mmol/L, creatinine 44.7 μmol/L, glucose 4.5 mmol/L, and bilirubin 4.8 μmol/L. Daily urinary protein excretion ranged from 0.2475 g/day to 1.32 g/day. The eGFR, calculated using the Schwartz formula, was 129 mL/min/1.73 m^2^. Patient 1 was started on nephroprotective therapy with Ramipril at a dose of 2.5 mg/day with blood pressure monitoring.

Patient 2, the older sibling, is a 13-year-old male. The disease manifested at the age of 1.5 years. Initial symptoms included microscopic hematuria (25–30 erythrocytes per high-power field) detected during an episode of obstructive bronchitis. From the age of 3 years, episodes of macroscopic hematuria were observed. The patient is also presented with significant proteinuria (2.3 g/L). The preliminary diagnosis was unclassified hereditary nephropathy. Sensorineural hearing loss was not detected; however, ophthalmologic examination revealed retinal angiopathy. Renal ultrasonography showed no structural abnormalities. Renal biopsy demonstrated focal global and segmental glomerulosclerosis with mild interstitial fibrosis and tubular atrophy (grade 1). Electron microscopy findings were consistent with a hereditary disorder of type IV collagen.

Laboratory examination revealed hemoglobin 123 g/L, erythrocytes 4.36 × 10^12^/L, platelets 341 × 10^9^/L, leukocytes 7.71 × 10^9^/L, and ESR 10 mm/h. Urinalysis demonstrated persistent proteinuria (up to 2.31 g/L) and hematuria (15–20 erythrocytes per high-power field). The specific gravity was 1.002, and the pH was 7.0. Biochemical blood analysis showed total protein 49 g/L, ALT 7.6 U/L, AST 19.6 U/L, urea 8.2 mmol/L, creatinine 69 μmol/L (previously 46.8 μmol/L), glucose 4.6 mmol/L, and bilirubin 3 μmol/L. Daily urinary protein excretion ranged from 0.436 to 1.815 g/day. The estimated glomerular filtration rate (eGFR) calculated by the Schwartz formula was 82 mL/min/1.73 m^2^, with a subsequent decrease to 47 mL/min/1.73 m^2^. At the age of 12 years, the patient was diagnosed with chronic kidney disease stage G3A3. Patient 2 was advised to increase the dose of Ramipril from 5 mg/day to 20 mg/day with blood pressure monitoring 2–3 times daily.

WES analysis was performed on the patients. To prepare standard exome sequencing libraries, the SureSelect V6-Post kit (Agilent Technologies, Santa Clara, CA, USA) was used to construct a paired-end sequencing library on the Illumina platform, using 1 μg of genomic DNA as starting material. Whole-Exome Sequencing (WES) was performed on a NovaSeq 6000 platform (Illumina Inc., San Diego, CA, USA) according to the manufacturer’s instructions. Exome sequencing services were provided by Macrogen. Bioinformatics analysis of the sequencing data was performed using a standard high-throughput pipeline. Raw reads were aligned to the hg38 reference genome. Genetic variants were identified using the Genome Analysis Toolkit (GATK) v4.5.0.0 and annotated via SnpEff. Variant filtering and clinical prioritization were conducted using dbSNP, the 1000 Genomes Project, ESP6500, dbNSFP, ClinVar, and the ACMG guidelines [10].

Key summary statistics of the raw sequence data obtained from the samples are presented in Table 1.

Detailed alignment scores for each sample, depth, coverage percentage, and variant scores for all samples are given in Table 2.

Data filtering revealed the variants in the *COL4A3*, *COL4A4*, *COL4A5*, and *COL4A6* genes presented in Supplementary Table S1. Thus, WES analysis identified a previously reported but insufficiently characterized genetic variant (c.1588-2A>G) in intron 23 of the *COL4A5* gene (NM_033380.2). The other variants identified in the priority genes were benign (Supplementary Table S1).

Variant was validated by Sanger sequencing using the BigDyeTerminator© v3.1 cycle sequencing kit (Applied Biosystems, Waltham, MA, USA) and a 3730 XL sequencer (Applied Biosystems, Inc.), following the manufacturer’s guidelines. The following primer sequences were used: forward (5’-GTACTTTGTTTGATTCCTTGACTC-3’) and reverse (5’-ATATCAAACCAACTCACAGGC-3’). The product length is approximately 402 bp.

**Figure 2:**
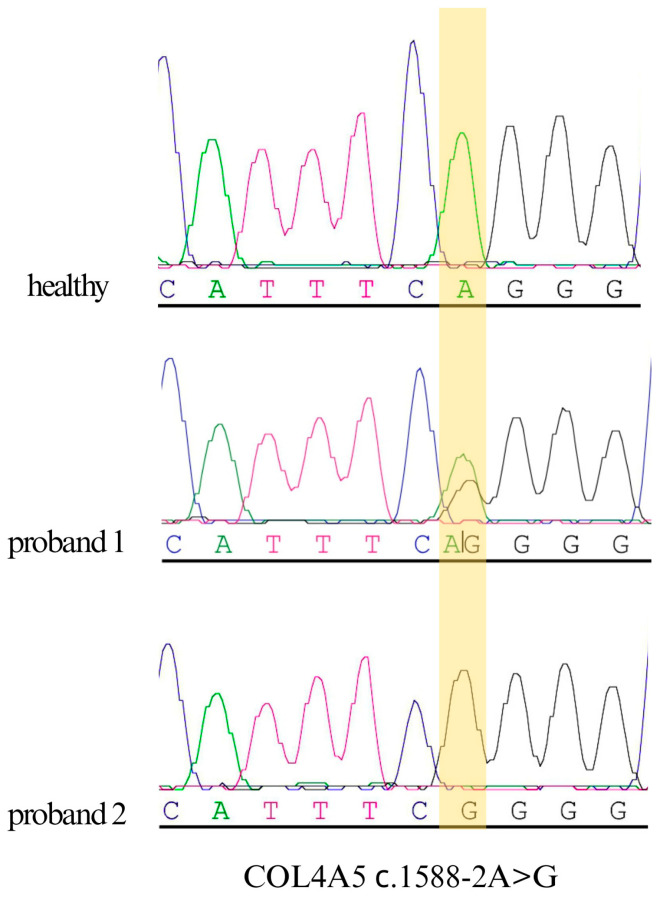
Sanger sequencing confirmation of the identified intronic *COL4A5* variant c.1588-2A>G. Electropherograms demonstrate a heterozygous A>G substitution in proband 1 and a hemizygous variant in proband 2. The variant position is highlighted.

Sanger sequencing was conducted on DNA samples from siblings and a conditionally healthy individual to confirm the presence of the c.1588-2A>G variant (Figure 2). The results indicate that the brother is a hemizygous carrier of the c.1588-2A>G variant, whereas the sister is a heterozygous carrier.

**Figure 3:**
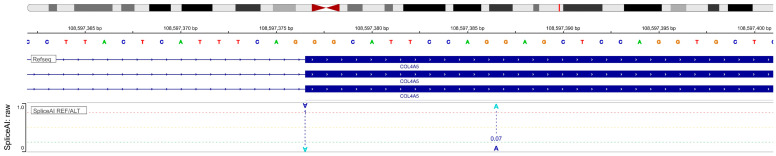
SpliceAI prediction results for the *COL4A5* c.1588-2A>G variant. The upper track represents the genomic coordinates of chromosome X surrounding the variant position. The middle tracks display the annotated *COL4A5* transcripts. The lower track shows SpliceAI REF/ALT predictions for the reference (dark blue) and alternate sequences (light blue) within the analyzed region. “A” and “D” symbols indicate predicted splice acceptor and donor sites, respectively. Numerical labels represent the corresponding SpliceAI scores at the variant position.

To predict the potential impact of intron variant, the bioinformatics tool SpliceAI (https://spliceailookup.broadinstitute.org/, accessed on 30 January 2026) was used. According to the in silico analysis, the splice acceptor site showed a splicing change, ranging from 1 to 0.9 before and after the variant. These results demonstrate a high probability of disruption of normal splicing (Figure 3).

The c.1588-2A>G variant demonstrates the highest acceptor loss (AL = 1.00), indicating possibility of a near-complete loss of canonical splice acceptor site function. The reference (REF) score for this site is 1.00, whereas the alternative (ALT) score decreases to 0.00, indicating possibility of a complete loss of the variant sequence recognition by the spliceosome. Additionally, a high probability of alternative acceptor site formation (acceptor gain, AG = 0.90) was observed. This event is characterized by a sharp increase in the ALT score (0.97) compared with the REF score (0.07), indicating possible activation of the cryptic splice site in the variant sequence. The effect of this variant on donor sites appears to be minor: the donor loss is 0.03, and the donor gain is 0.01, indicating no significant changes in donor splicing signals. In summary, the combined data suggest that the c.1588-2A>G variant may result in disruption of normal splicing through loss of the canonical acceptor site and possible activation of a cryptic acceptor site. These data suggest that the c.1588-2A>G variant may affect splicing and lead to a frameshift, potentially resulting in a premature termination codon.

The I-TASSER software (version 5.2) was used to predict the structural organization of both the wild-type COL4A5 protein and the protein containing the c.1588-2A>G variant. The wild-type amino acid and cDNA sequences of the *COL4A5* gene were obtained from the UCSC Genome Browser (http://genome.ucsc.edu/, accessed on 30 January 2026). The altered amino acid sequence of COL4A5 was generated by mapping the variant on the cDNA using SnapGene 8.2.2 software. Then, amino acids 1–1500 of both the wild-type and altered *COL4A5* sequences were modeled using the I-TASSER server (https://aideepmed.com/I-TASSER/, accessed on 30 January 2026) [11]. The quality metrics for the generated models are presented in Table 3.

**Figure 4:**
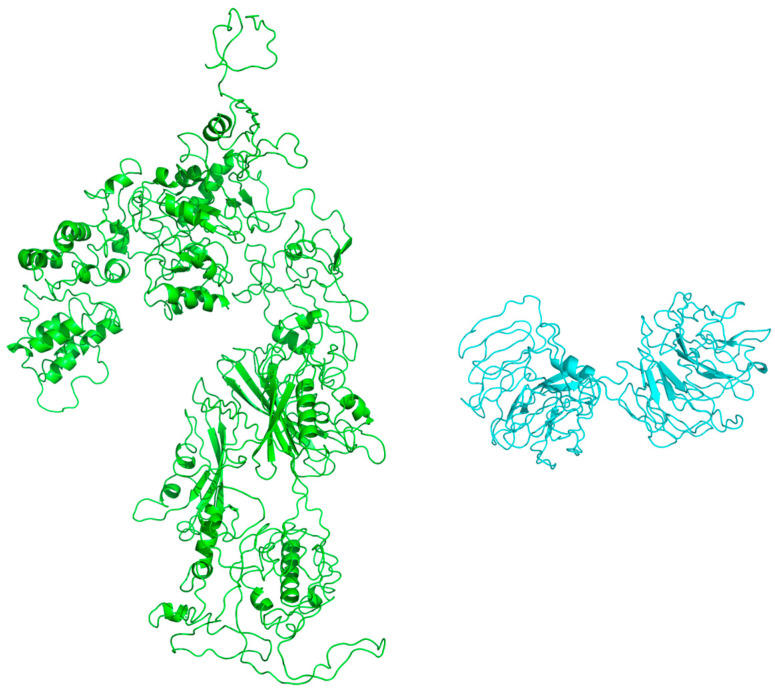
Predicted 3D structures of wild-type (**left**) and variant containing (**right**) *COL4A5* proteins. The variant protein exhibits significant conformational changes relative to the wild type.

The 3D structure of the protein was visualized using PyMol 3.1 software. Comparative modeling of the 3D structures revealed a significant change in the altered protein, primarily due to a nearly threefold reduction in the amino acid sequence (Figure 4). This substantial reduction likely disrupts the normal spatial conformation of the protein and impairs its biological function.

The interpretation of the *COL4A5* c.1588-2A>G variant was based on evidence according to ACMG/AMP criteria. PVS1 was assigned due to its location at a canonical splice acceptor site. However, in the absence of functional RNA data, the PVS1 criterion was cautiously applied at a Strong level (PVS1_Strong). PP1 was supported by segregation of the variant with the disease phenotype within the family. PP3 was based on SpliceAI predictions, indicating a potential impact on RNA splicing. PP4 was supported by the presence of a highly specific clinical phenotype, including hematuria, proteinuria, and characteristic renal biopsy findings consistent with a *COL4A5*-associated disorder. These combined criteria support the classification of the variant as likely pathogenic according to the ACMG/AMP guidelines (PVS1_Strong+PP1+PP3+PP4) [12], while definitive confirmation of its pathogenic effect requires experimental functional validation. Overall, the combined clinical, histopathological, and genetic findings support the conclusion that the identified *COL4A5* splice-site variant is likely responsible for the development of AS in the reported patients.

## 3. Discussion

Pathogenic variants in the *COL4A3*, *COL4A4*, and *COL4A5* genes affect the assembly of the type IV collagen network in basement membranes, resulting in Alport syndrome [13]. While exon variants are most frequently reported, increasing evidence indicates that both canonical and deep intronic variants represent a significant and understudied cause of the disease [8].

The most extensively studied pathogenic variants are those occurring in the canonical splice sites, specifically the donor (GT) and acceptor (AG) splice sites, which are located at positions ±1–2 relative to the exon–intron boundary. These variants are typically classified as pathogenic due to their significant impact on splicing. However, a rare exception exists for the canonical dinucleotide splice site type, such as GC/AG, which accounts for less than 1% of cases. Variants at these sites do not always result in exon skipping; instead, they may lead to partial deletion of exons or exonization of introns. This distinction is crucial for assessing renal prognosis when evaluating in-frame or out-of-frame deletions at the transcript level [2].

A frequent consequence of variants in canonical splice sites is the complete skipping of an adjacent exon. For example, the novel c.834+2T>G variant in the *COL4A5* donor site has been demonstrated to cause complete skipping of exon 14. This leads to an in-frame deletion of 18 amino acids in the Gly-Xaa-Yaa repeat region of the α5(IV) chain, which is likely to produce a truncated or unstable protein [14]. Similarly, in *COL4A3*, the synonymous variant c.765G>A (p.(Thr255Thr)) at the last nucleotide of exon 13 induces exon skipping, leading to an in-frame deletion of 28 amino acids. This molecular mechanism is linked to the slowly progressive autosomal dominant form of Alport syndrome [15]. In *COL4A5*, the c.2917+1G>C variant was observed to cause exon 33 skipping, while another substitution at the same location (G>A) primarily activates the cryptic splice site [16]. Other cases of exon skipping include skipping of exon 50 in *COL4A5* due to the c.4529-2A>T [17] variant and skipping of exon 48 due to the c.4688+2T>C variant [18].

This study describes a familial case of AS associated with the intronic *COL4A5* variant c.1588-2A>G. Two affected siblings were examined, with disease onset occurring at 1.5 years of age in the brother and 6 years of age in the sister. Family history was suggestive of X-linked inheritance, as the patients’ mother has hematuria and their maternal uncle reportedly had kidney disease. Nevertheless, detailed clinical records and molecular genetic data for the mother and other family members were unavailable at the time of the study, limiting assessment of the broader familial phenotype and variant segregation.

WES has not found any pathogenic or likely pathogenic variants in the exon regions of *COL4A3*, *COL4A4*, *COL4A5*, or *COL4A6* according to ClinVar annotations and ACMG/AMP criteria in our probands. In silico analysis of the c.1588-2A>G variant revealed a probable loss of the canonical acceptor site and simultaneous activation of the cryptic acceptor site, potentially leading to a frameshift and presumed formation of a premature stop codon. The resulting transcript is likely to undergo nonsense-mediated mRNA decay [19]; however, even if translation occurs, the predicted protein would likely be nonfunctional. However, RNA-based functional studies were not performed in the present study; therefore, the exact splicing consequences of this variant remain to be experimentally confirmed. Accordingly, the structural alterations of the *COL4A5* protein predicted using I-TASSER represent a computational model that has not been validated experimentally.

In the ClinVar database, the c.1588-2A>G variant is described as causing protein structure disruption [20]. However, this evidence is based on RNA sequencing data in the context of thyroid cancer and does not have relation with AS or its symptoms [21]. This variant was added to the database in November 2025; therefore, it was not identified during the initial WES data analysis, which was conducted using the ClinVar version dated 16 July 2024.

The Leiden Open Variation Database (LOVD) is a web-based open-source database that represents a collection of variants found in individuals. The LOVD database provides an open-access platform for the submission and sharing of gene variant data [22]. Therefore, ClinVar is more reliable for clinical variant interpretation, whereas LOVD provides more detailed gene-specific information but with less standardized curation. The variant c.1588-2A>G is also registered in the LOVD (version 3.0) database as pathogenic [23]. The individual in whom the variant was found is a male diagnosed with end-stage renal disease (ESRD) at age 19 and confirmed Alport syndrome at age 23 [24]. There weren’t any publications describing this case and/or the variant in the available scientific literature. Due to the lack of detailed clinical and phenotypic information, this case alone does not provide sufficient evidence to establish or support the genotype–phenotype correlation. The present case provides additional clinical context that is consistent with the previously reported pathogenic classification of the c.1588-2A>G variant and helps to further clarify its associated phenotype.

According to clinical symptoms, the identified variant is most likely the cause of Alport syndrome in two patients. Both patients exhibited characteristic features of Alport syndrome, including hematuria and proteinuria. The male patient had an earlier onset and a more severe clinical course than his sister, consistent with the known X-linked inheritance pattern. Furthermore, histopathological findings, including focal segmental glomerulosclerosis and ultrastructural changes in the glomerular basement membrane, are consistent with defects in type IV collagen. Based on clinical history, genetic analysis results and existing evidence, it can be concluded that the c.1588-2A>G variant is highly probable to be the underlying cause of Alport syndrome in this family. However, due to the limitations of the present study, including the lack of functional RNA-based validation, a definitive pathogenic effect cannot be established. Based on the available clinical, genetic, and in silico evidence, the *COL4A5* c.1588-2A>G variant is classified as likely pathogenic.

It should be noted that this study did not analyze deep intronic variants, which are not detected by WES and could potentially lead to splicing abnormalities and, consequently, alter protein structure and function. Furthermore, RNA analysis was not performed to confirm the presence of aberrant splicing and the functional consequences of the identified variant.

## 4. Conclusions

Our study describes the *COL4A5* c.1588-2A>G splice-site variant in a Kazakhstani family with clinical features consistent with X-linked Alport Syndrome. The combined clinical presentation, familial segregation pattern, and in silico analyses support a likely pathogenic role of this variant and suggest a potential disruptive effect on normal splicing. The phenotypic differences observed between the affected siblings are also consistent with the known variability of X-linked disease expression. This report expands the variant spectrum of *COL4A5* and underscores the importance of evaluating canonical splice-site variants in pediatric patients with hereditary nephropathy to ensure early diagnosis and appropriate clinical management.

## Figures and Tables

**Table 1:** Fastq statistics.

Sample Name	Total Yield (bp)	Total Reads	GC (%)	AT (%)	Q20 (%)	Q30 (%)
Patient 1	8,086,480,954	53,552,854	50.79	49.21	98.89	95.87
Patient 2	8,012,671,852	53,064,052	50.45	49.55	98.98	96.03

Total Yield (bp)—total number of bases sequenced; Total Reads—total number of reads; GC (%)—GC content; AT (%)—AT content; Q20 (%)—ratio of bases that have a Phred quality score of over 20; Q30 (%)—ratio of bases that have a Phred quality score of over 30.

**Table 2:** Number of reads, coverage, and variant statistics by sample.

Sample Name	Patient 1	Patient 2
Total reads	53,552,760	53,063,934
Average length (bp)	146.55	149.28
Number of on-target genotypes (≥1×)	36,378,510	36,432,945
% Coverage of target regions (≥10×)	99.7	99.8
% Coverage of target regions (≥30×)	99.2	99.2
% Coverage of target regions (≥50×)	99.4	95.4
Number of SNPs	76,465	76,926
Missense Variants	11,838	11,758
Stop Gained	106	117
Stop Lost	26	24
Number of INDEL	14,789	15,127
Frameshift Variants	261	256
% Found in dbSNP156	99.4	99.3

**Table 3:** The models’ quality metrics.

Name	Model of WT Protein	Model of Variant Containing Protein
C-score	−0.24	0.68
Estimated TM-score	0.68 + −0.12	0.81 + −0.09
Estimated RMSD	10.4 + −4.6	6.0 + −3.7
No. of decoys	600	1200
Cluster density	0.1486	0.5000

C-score—confidence score; TM-score—template modeling score; RMSD—root mean square deviation.

## Data Availability

The data that support the findings of this study are available from the corresponding author upon reasonable request.

## Supplementary Materials

The following supporting information can be downloaded at: https://www.mdpi.com/article/10.3390/cimb48060588/s1.

## Abbreviations

The following abbreviations are used in this manuscript:

AS	Alport syndrome
XLAS	X-linked Alport syndrome
CSSVs	Canonical splice-site variants
WES	Whole-Exome Sequencing
ACMG	American College of Medical Genetics and Genomics
ALT	Alanine transaminase
AST	Aspartate transaminase
eGFR	Estimated glomerular filtration rate
AL	Canonical acceptor site
AG	Acceptor gain
